# The expression and role of TRPV2 in esophageal squamous cell carcinoma

**DOI:** 10.1038/s41598-019-52227-0

**Published:** 2019-11-05

**Authors:** Michihiro Kudou, Atsushi Shiozaki, Yuzo Yamazato, Keita Katsurahara, Toshiyuki Kosuga, Katsutoshi Shoda, Tomohiro Arita, Hirotaka Konishi, Shuhei Komatsu, Takeshi Kubota, Hitoshi Fujiwara, Kazuma Okamoto, Mitsuo Kishimoto, Eiichi Konishi, Yoshinori Marunaka, Eigo Otsuji

**Affiliations:** 1 Division of Digestive Surgery, Department of Surgery, Kyoto Prefectural University of Medicine, Kyoto, 602-8566 Japan; 2 Department of Pathology, Kyoto Prefectural University of Medicine, Kyoto, 602-8566 Japan; 3 Department of Molecular Cell Physiology, Graduate School of Medical Science, Kyoto Prefectural University of Medicine, Kyoto, 602-8566 Japan; 4 Research Center for Drug Discovery and Pharmaceutical Development Science, Research Organization of Science and Technology, Ritsumeikan University, Kusatsu, 525-8577 Japan; 5 Research Institute for Clinical Physiology, Kyoto Industrial Health Association, Kyoto, 604-8472 Japan

**Keywords:** Cancer, Cell biology, Molecular biology

## Abstract

Background: Transient receptor potential vanilloid 2 (TRPV2) was recently shown to be involved in migrant potentials. The present study aimed to investigate its role in esophageal squamous cell carcinoma (ESCC). Methods: Knockdown experiments were conducted using TRPV2 siRNA in human ESCC cell lines, and anti-tumor effects were analyzed. The gene expression profiles of cells were analyzed using a microarray method. An immunohistochemical staining was performed on 62 primary tumor samples. Results: TRPV2 overexpression was observed in TE15 and KYSE170 cells. TRPV2 depletion suppressed proliferation, cell cycle progression, and invasion/migration ability, and induced apoptosis. A pathway analysis of microarray data showed that TRPV2 depletion down-regulated WNT/β-catenin signaling-related genes and basal cell carcinoma signaling-related genes. The suppression of tumor functions, such as proliferation, invasion, and angiogenesis, was predicted in the ontology analysis. Immunohistochemical analysis revealed a correlation between strong TRPV2 expression and a poor prognosis in ESCC patients. Conclusion: The present results suggest that TRPV2 regulates cancer progression by affecting WNT/β-catenin or basal cell carcinoma signaling, and that TRPV2 strong expression is associated with a worse prognosis in ESCC patients. These results provide an insight into the role of TRPV2 as a novel therapeutic target or biomarker for ESCC.

## Introduction

Transient receptor potential vanilloid (TRPV) channels have been identified as one of the molecules activated by capsaicin, a vanilloid-like molecule. TRPV1 was initially reported as a membrane protein triggered by heat or pain stimuli, VR1 in 1997^1^. Five additional molecules of the subfamily were subsequently cloned and named TRPV2, TRPV3, TRPV4, TRPV5, and TRPV6^2–6^. Although all members of the TRPV subfamily were initially presumed to have similar functions, such as heat sensors, extensive physiological studies later revealed that TRPV2–6 did not all respond to temperature stimuli. Moreover, TRPV1–4 channels have non-selective cation-conducting pores, while the pores of TRPV5 and TRPV6 are highly calcium selective^7^. Among members of the TRPV subfamily, TRPV2 is expressed in many organs, such as the digestive tract, pancreas, and liver; furthermore, TRPV2 has been implicated in bowel inflammation, intestinal peristalsis, and the autocrine effects of insulin on pancreatic β-cells^8–10^. Therefore, TRPV2 may be a therapeutic target of digestive diseases.

Previous studies reported that TRPV2 was involved in cancer progression, migration, and invasion as well as in the therapeutic effects of anticancer drugs. Gambade *et al*. showed that the activation of TPRV2 regulated cancer migration via calcium entry^11^. Similarly, the involvement of TRPV2 in migration and invasion was demonstrated in bladder and prostate cancers^12,13^. TRPV2 was identified as a poor prognostic marker in gastric cancer and, in contrast, a good prognostic marker in breast cancer^14,15^. Liberati *et al*. interpreted this discrepancy as TRPV2 playing a major role in initial events that parallel the transformation of a single normal stem/progenitor cell into a tumor cell, while the expression of TRPV2 was modulated during carcinogenesis that followed long-term and continuous exposure to different carcinogenetic agents, resulting in tumor growth, progression, and the selection of more aggressive tumor clones^16^. We demonstrated that TRPV2 was overexpressed in cancer stem cells derived from ESCC, and suggested the potential of tranilast, a TRPV2-specific inhibitor, as a therapeutic agent for cancer stem cells^17^. Although alterations in TRPV2 expression in cancer cells or the involvement of TRPV2 in cancer functions have been reported in several types of carcinomas including esophageal squamous cell carcinoma (ESCC)^18^, the oncological and physiological roles of TRPV2 in ESCC have remained unclear. The elucidation of these roles is needed to develop TRPV2-targeted therapies for ESCC.

Hence, we investigated the role of TPRV2 in ESCC using a cancer function assay with the knockdown of TRPV2 by siRNA, microarray, pathway, and gene ontology analyses. The significance of TRPV2 expression in ESCC samples was then evaluated by immunohistochemical staining. The present study aimed to clarify the role of TRPV2 and its clinical significance in ESCC.

## Materials and Methods

All methods, especially the experiments using human samples, were carried out in accordance with relevant guidelines^19^.

### Ethical approval and consent to participate

This study was approved by the Research Ethics Committee of the Kyoto Prefectural University of Medicine (No. ERB-C-1178). Comprehensive informed consent for use of clinical data and samples was obtained from all eligible patients.

### Cell culture, antibodies, and other materials

The human ESCC cell line TE5, TE8, TE19m and TE15 was obtained from the Cell Resource Centre for Biomedical Research at the Institute of Development, Aging, and Cancer (Tohoku University, Sendai, Japan). The human ESCC cell lineLYSE150 and KYSE170 was obtained from the Japanese Collection of Research Bioresources Cell Bank (Osaka, Japan). The cells were cultivated using our previously reported protocols^20^. These cell lines were grown in RPMI-1640 medium (Nacalai Tesque, Kyoto, Japan) supplemented with 100 U/mL penicillin, 100 μg/mL streptomycin, and 10% fetal bovine serum (FBS). Cells were cultured in flasks and dishes in a humidified incubator at 37 °C in 5% CO_2_ in air. A rabbit monoclonal anti-TRPV2 antibody was used for the immunohistochemical analysis, and a protein assay was obtained from Santa Cruz Biotechnology (Santa Cruz, CA, USA). The following antibodies were used in the Western blotting analysis: a rabbit monoclonal anti-caspase 3 antibody and rabbit monoclonal anti-cleaved caspase 3 antibody, which were purchased from Cell Signaling Technology (Beverly, MA). A mouse monoclonal anti-β-actin antibody was purchased from Sigma-Aldrich (St. Louis, MO, USA).

### Western blotting

Western blotting was performed using our previously reported protocols^20^. Briefly, cells were harvested in M-PER lysis buffer (Pierce, Rockford, IL) supplemented with protease inhibitors (Pierce, Rockford, IL). Protein concentrations were measured with a modified Bradford assay (Bio-Rad, Hercules, CA). Cell lysates containing equal amounts of total protein were separated by SDS-PAGE and then transferred onto PVDF membranes (GE Healthcare, Piscataway, NJ). These membranes were then probed with the indicated antibodies, and proteins were detected using an ECL Plus Western Blotting Detection System (GE Healthcare, Piscataway, NJ).

### Small interfering RNA (siRNA) transfection

SiRNA transfection was carried out using our previously reported protocols^20^. Cells were transfected with 20 nmol/L TRPV2 siRNA (Stealth RNAi siRNA #HSS122144, Invitrogen, Carlsbad, CA) using Lipofectamine RNAiMAX reagent (Invitrogen) in accordance with the manufacturer’s instructions. Medium containing siRNA was replaced with fresh medium after 24 h. Control siRNA (Stealth RNAi siRNA Negative Control; Invitrogen) was used as a negative control.

### Cell proliferation assay

Cell proliferation assay was similarly performed using our previously reported protocols^20^.

Cells were seeded on 6-well plates at a density of 1.5 × 10^5^ cells per well for TE15 and 0.4 × 10^5^ cells per well for KYSE170, and incubated at 37 °C with 5% CO_2_. siRNA was transfected 24 h after cells had been seeded. Cells were detached from flasks with trypsin-EDTA 48 and 72 h after siRNA transfection and were counted using a hemocytometer.

### Cell cycle analysis

Cell cycle progression was observed using our previously reported protocols^20^. In TRPV2 knockdown experiments, cell cycle progression was evaluated 72 h after siRNA transfection using FACS. Briefly, cells were treated with Triton X-100, and cell nuclei were stained with PI RNase staining buffer (Becton-Dickinson Biosciences, San Jose, CA, USA). The DNA content was then measured using a Becton-Dickinson Accuri C6 (Becton-Dickinson Biosciences). At least 10,000 cells were counted, and BD Accuri C6 software was used to analyze the cell cycle distribution.

### Analysis of apoptotic cells

Cell apoptosis was evaluated using our previously reported protocols^20^. Cells were harvested 72 h after siRNA transfection and stained with fluorescein isothiocyanate (FITC)- conjugated Annexin V and propidium iodide (PI) using an Annexin V-FITC kit (Beckman Coulter, Brea, CA) in accordance with the manufacturer’s protocol. The proportion of apoptotic cells was analyzed by fluorescence-activated cell scoring (FACS) using a BD Accuri C6 (BD Biosciences).

### Real-time reverse transcription-polymerase chain reaction (RT-PCR)

Gene expression was assessed by our previously reported RT-PCT method^20^. Total RNA was extracted using a RNeasy kit (Qiagen, Valencia, CA). mRNA expression levels were measured by quantitative real -time PCR (7300 Real-Time PCR System; Applied Biosystems, Foster City, CA) using TaqMan Gene Expression Assays (Applied Biosystems) in accordance with the manufacturer’s instructions. Expression levels were measured for the following genes: TRPV2 (Hs00901640_m1), WNT10A(Hs00228741_m1), TGFβ2(Hs00234244_m1), TGFβ2R(Hs00559661_m1), and Gli1 (Hs00171790_m1), Snai1 (Hs00195591_m1), Zeb2 (Hs00207691_m1), CDH2 (Hs00983056_m1), CD44 (Hs00153304_m1), SOX2 (Hs01053049_m1) (Applied Biosystems). Gene expression was normalized to the housekeeping gene β-actin (Hs01060665 g1; Applied Biosystems). Assays were performed in triplicate.

### Analysis of cell migration and invasion

The migration assay was conducted using a Cell Culture Insert with a pore size of 8 μm (BD Biosciences, Bedford, MA, USA) by our previously reported protocols^20^. Biocoat Matrigel (BD Biosciences) was used to evaluate cell invasion potential. Cells (TE15: 3.25 × 10^5^ cells per well, KYSE170: 0.6 × 10^5^ cells per well) were seeded in the upper chamber in serum-free medium 24 h after siRNA transfection. The lower chamber contained medium with 10% FBS. The chambers were incubated at 37 °C for 48 h in 5% CO_2_, and non-migrating or non-invading cells were then removed from the upper side of the membrane by scrubbing with cotton swabs. Migrating or invading cells were fixed on the membrane and stained with Diff-Quick staining reagents (Sysmex, Kobe, Japan). The migrating or invading cells on the lower side of the membrane were counted in four independent fields of view at ×100 magnification for each insert. Each assay was performed in triplicate.

### Microarray sample preparation and hybridization

Microarray analysis was conducted using our previously reported protocols^20^. Briefly, total RNA was extracted using a RNeasy kit (Qiagen). RNA quality was monitored with an Agilent 2100 Bioanalyzer (Agilent Technologies, Santa Clara, CA). Cyanine-3 (Cy3)-labeled cRNA was prepared from 0.1 μg of total RNA using a Low Input Quick Amp Labeling Kit (Agilent) in accordance with the manufacturer’s instructions. Samples were purified using RNeasy columns (Qiagen). A total of 0.60 μg of Cy3-labeled RNA was fragmented and hybridized to an Agilent SurePrintG3 Human Gene Expression 8 × 60 K ver3.0 Microarray for 17 h. Slides were washed and scanned immediately using an Agilent DNA Microarray Scanner (G2565CA) in the one color scan setting for 8 × 60 K array slides.

### Processing of microarray data

Processing of microarray data was conducted using our previously reported protocols^20^. Scanned images were evaluated with Feature Extraction Software 10.10 (Agilent) using default parameters to acquire background-subtracted and spatially detrended processed signal intensities^20^. Signal transduction networks were subsequently analyzed using an Ingenuity Pathway Analysis (IPA) (Ingenuity Systems, Qiagen, Redwood City, CA)^20^.

### Patients and primary tissue samples

ESCC human samples were obtained from 62 patients with histologically confirmed primary ESCC undergoing esophagectomy at Kyoto Prefectural University of Medicine between 1999 and 2009. All of them were embedded in paraffin after 12 h of formalin fixation just after the surgery. We excluded patients with non-curative resected tumors, synchronous or metachronous cancers (in addition to ESCC), preoperative chemotherapy, or radiation therapy. All eligible patients provided written informed consent. The data of relevant clinicopathological and survival were obtained from the hospital database. Staging was principally diagnosed based on the International Union Against Cancer/Tumor Node metastasis Classification of Malignant Tumors (7^th^ edition)^21^.

### Immunohistochemistry

TRPV2 protein expressions in human ESCC tissues were evaluated by the same immunohistochemical staining protocol as our previous study^20^. Paraffin sections of tumor tissues (thickness of 4 μm) were subjected to immunohistochemical staining using the avidin-biotin-peroxidase method^20^. Briefly, paraffin sections were dewaxed with xylene and hydrated with a graded series of alcohol^20^. Endogenous peroxidases were quenched by incubating the sections for 30 min in 0.3% H_2_ O_2_^20^. The Avidin/Biotin Blocking Kit (Vector Laboratories, Burlingame, CA) was used for the blocking of endogenous biotin, biotin receptors, and avidin-binding sites^20^. Sections were then treated with a protein blocker and incubated at 4 °C overnight with the primary antibody^20^. The avidin-biotin-peroxidase complex (Vectastain ABC Elite kit; Vector Laboratories, Burlingame, CA) was visualized using diaminobenzidine tetrahydrochloride^20^. Sections were counterstained with hematoxylin^20^. These sections were then dehydrated through a graded series of alcohols, cleared in xylene, and mounted^20^. Immunohistochemical samples stained with TRPV2 were graded based on staining intensities, strong or weak expression, and proportions^20^.

### Statistical analysis

Statistical analysis was performed using same method of our previous study^20^. Fisher’s exact test was used to evaluate differences between proportions, and the Student’s *t*-test was employed to evaluate continuous variables^20^. Survival curves were constructed using the Kaplan–Meier method, and differences in survival were examined using the Log-rank test^20^. A multivariate analysis of the factors influencing survival was performed using Cox’s proportional hazard model^20^. Differences were considered to be significant when the relevant p value was <0.05^20^. These analyses were performed using JMP statistical software (version 12, SAS Institute Inc., Cary, NC)^20^.

## Results

### TRPV2 protein and mRNA expression in ESCC cell lines

**Figure 1:**
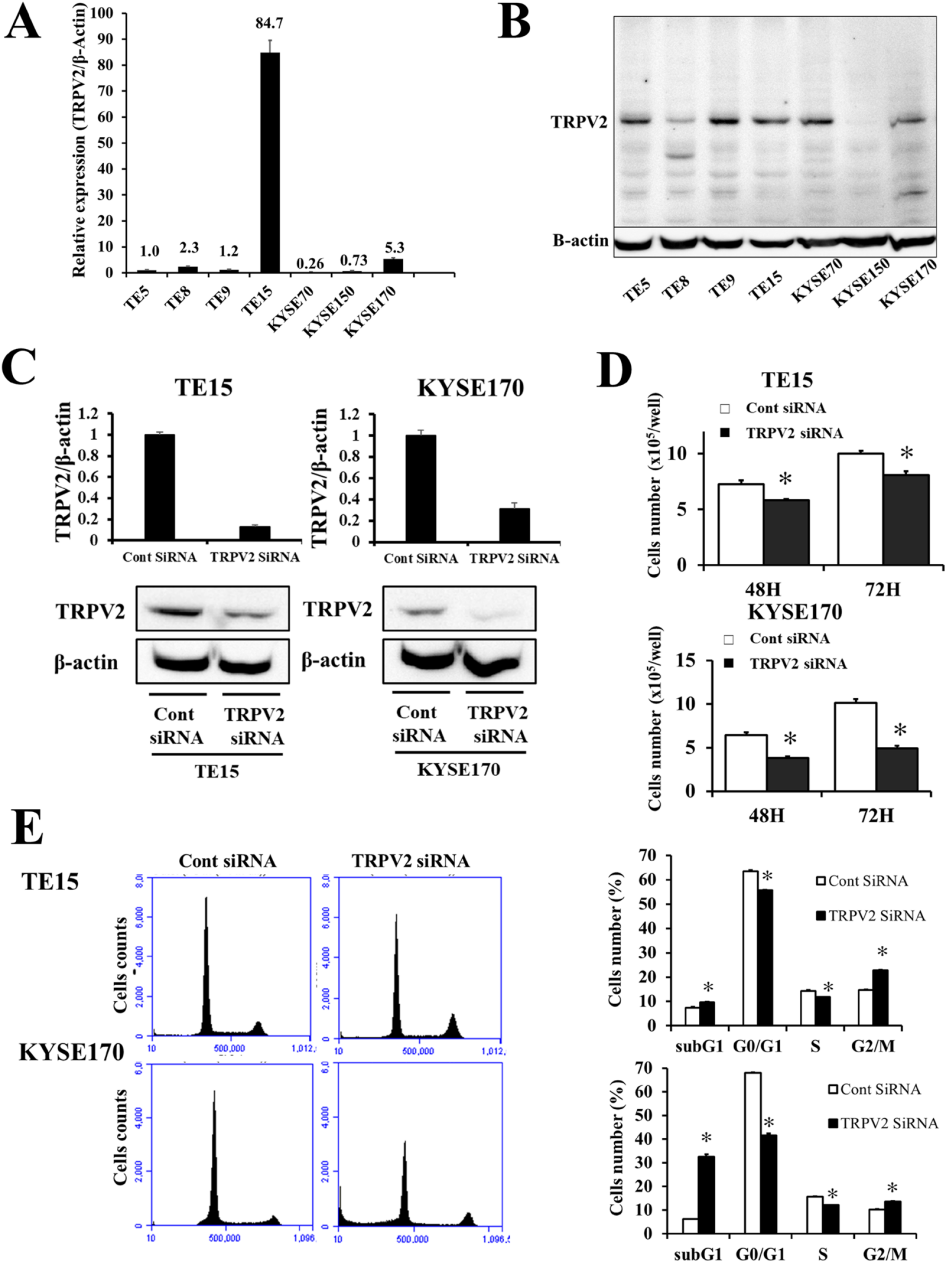
TRPV2 controls the proliferation and cell cycle progression of ESCC cells, (**A**) TRPV2 mRNA expression was analyzed in 7 ESCC cell lines. Quantitative RT-PCR showed that TRPV2 was strongly expressed in TE15, and more strongly expressed in KYSE170 than in the other ESCC cell lines. (**B**) TRPV2 protein expression of ESCC cell lines was evaluated by western blotting. Upper panel showed full-length gels of TRPV2, and lower panel indicated the cropped image of same gels for evaluating β-actin expression (full-length image: Supplementary Fig. 3). TRPV2 expression in TE15 and KYSE170 was similar in Western blotting. (**C**) TRPV2 expression of ESCC cell lines transfected with control and TRPV2 siRNA was evaluated by quantitative RT-PCR and western blotting. The blotting figure showed the cropped image of TRPV2 or β-Actin bands on same gels (full-length image: Supplementary Fig. 4). TRPV2 siRNA effectively reduced TRPV2 mRNA and protein levels in TE15 and KYSE170 cells. Mean ± SEM. n = 3. *p < 0.05 (significantly different from control siRNA). (**D**) The down-regulation of TRPV2 inhibited the proliferation of TE15 and KYSE170 cells. The number of cells was counted 48 and 72 h after siRNA transfection. Mean ± SEM. n = 4. *p < 0.05 (significantly different from control siRNA). (**E**) The down-regulation of TRPV2 partially reduced cell cycle progression from the G1 to S phase in TE15 and KYSE170 cells. The cell population of sub-G1 increased in TPRV2-depleted TE15 and KYSE170 cells. Cells transfected with control or NIS siRNA were stained with propidium iodide (PI) and analyzed by flow cytometry. Mean ± SEM. n = 3. *p < 0.05 (significantly different from control siRNA).

Quantitative RT-PCR and Western blotting were performed to evaluate TRPV2 expression in the human ESCC cell lines, TE5, TE8, TE9, TE15, KYSE70, LYSE150, and KYSE170 (Fig. 1A,B). TRPV2 mRNA was strongly expressed in TE15, and more strongly expressed in KYSE170 than in the other ESCC cell lines. Meanwhile, similar intensity of TRPV2 protein expression was observed in TE5, TE9, TE15, KYSE70 and KYSE170.

### siRNA knockdown of TRPV2 suppressed the proliferation of ESCC cells

We conducted knockdown experiments using siRNA in the TRPV2-overexpressing ESCC cell lines, TE15 and KYSE170, and evaluated the effects of TRPV2 depletion on cell proliferation. TRPV2 siRNA effectively decreased TRPV2 mRNA and protein expression in TE15 and KYSE170 (Fig. 1C). Cell proliferation was significantly less in TRPV2-depleted cells than in control-siRNA-treated cells (Fig. 1D).

### TRPV2 depletion inhibited cell cycle progression and induced apoptosis in ESCC cells

**Figure 2:**
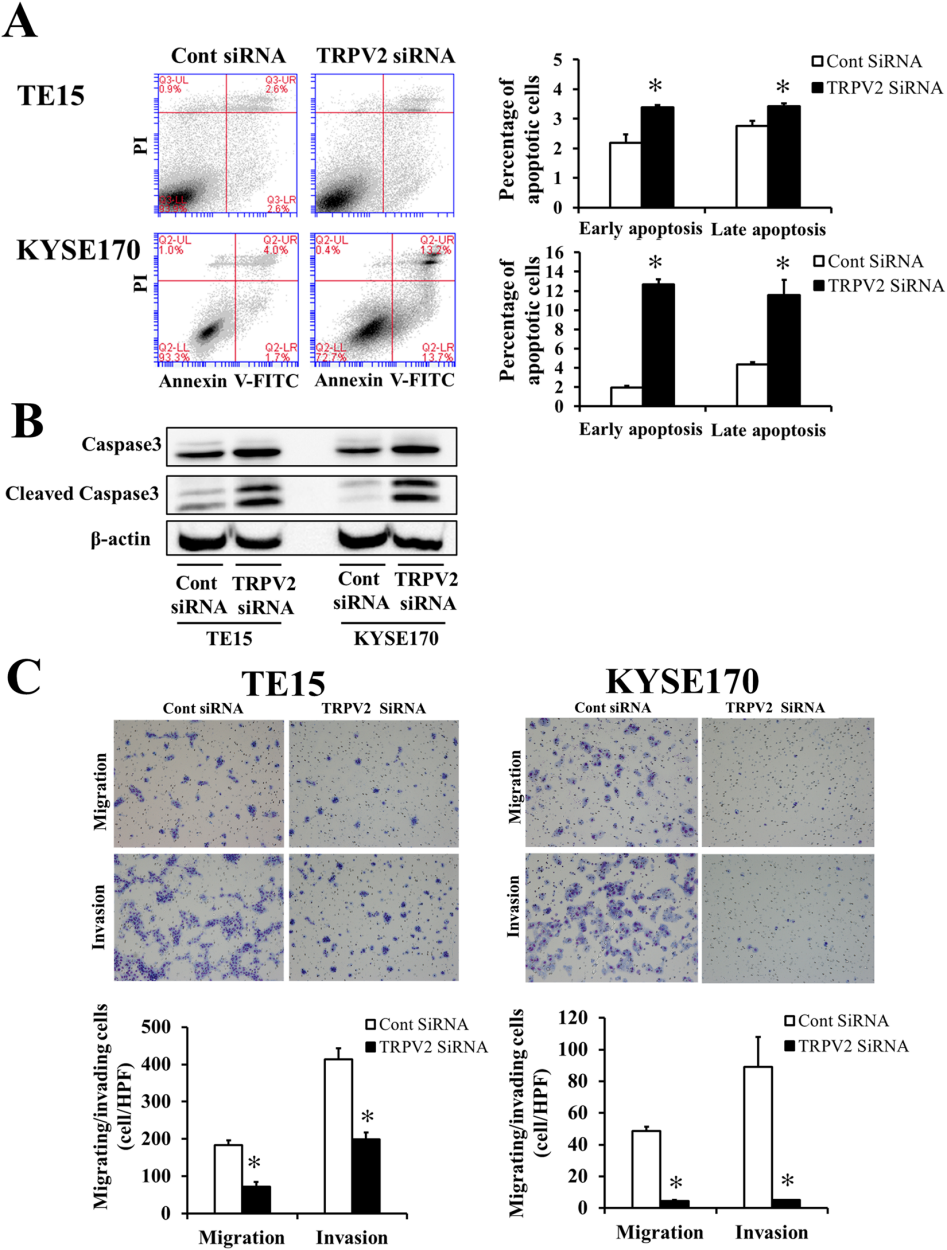
TRPV2 controls the survival, migration, and invasion of GC cells. (**A**) The down-regulation of TRPV2 induced early and late apoptosis in TE15 and KYSE170 cells. Apoptosis was assessed by flow cytometry using PI/annexin V double staining. Mean ± SEM. n = 3. *p < 0.05 (significantly different from control siRNA). (**B**) Apoptosis marker expression, caspase 3 and cleaved caspase 3, of ESCC cell lines transfected with control and TRPV2 siRNA was evaluated by western blotting. The western blotting figure indicated the clopped image of the bands on same gels (full-length image: Supplementary Fig. 5). The findings revealed that the expression of cleaved caspase 3, an apoptosis marker, was increased in TRPV2-depleted TE15 and KYSE170 cells. (**C**) The down-regulation of TRPV2 inhibited the migration and invasion of TE15 and KYSE170 cells. Cell migration and invasion were examined using the Boyden chamber assay. Mean ± SEM. n = 3. *p < 0.05 (significantly different from control siRNA).

A cell cycle analysis was performed to investigate the mechanisms underlying suppressed proliferation induced by TRPV2 depletion in ESCC cells (Fig. 1E). The transfection of TRPV2 siRNA partially inhibited cell cycle progression from the G1 to S phase in TE15 and KYSE170. Moreover, the cell population of sub-G1 increased in TPRV2-depleted cells, suggesting that TRPV2 depletion suppressed the proliferation of ESCC cells by inhibiting cell cycle progression or inducing apoptosis. In order to validate the induction of apoptosis by TRPV2 depletion, an apoptosis assay and Western blotting of the apoptosis markers, caspase 3 and cleaved caspase 3 were conducted. The results obtained revealed that the down-regulation of TRPV2 induced early apoptosis (annexin V-positive/PI negative) and late apoptosis (annexin V-positive/PI positive) in TE15 and KYSE170 72 hours after the siRNA knockdown (Fig. 2A). Western blotting showed that the expression of cleaved caspase 3 increased in TRPV2-depleted ESCC cells (Fig. 2B).

### TRPV2 controlled cell migration and invasion in ESCC cells

The effects of the down-regulation of TRPV2 on cell migration and invasion in ESCC cells were analyzed, and the results obtained revealed that the TRPV2 siRNA knockdown inhibited cell migration and invasion (Fig. 2C). These results indicated that TRPV2 plays an important role in the cancer cell migration and invasion of ESCC.

### Gene expression profiles of TRPV2-depleted ESCC cells

The gene expression profile of TRPV2-depleted KYSE170 cells was analyzed using microarrays and a bioinformatic study. Microarray results showed that the expression of 3697 genes displayed a fold change of >2.0 in KYSE170 cells upon the depletion of TRPV2. The number of up-regulated genes was 1372, while that of down-regulated genes was 2325. A list of 20 genes with expression levels that were the most strongly up- or down-regulated in TRPV2-depleted KYSE170 cells was shown in Supplementary Tables 1 and 2. The Ca^2+^-related genes, MICU1 and CACNB2, were strongly down-regulated in TRPV2-depleted KYSE170 cells. Furthermore, a gene ontology analysis (IPA) showed that the function of “the invasion of cells”, “migration of cells”, “migration of tumor cell lines”, “invasion of tumor cell lines”, “cell proliferation of tumor cell lines”, “apoptosis”, and “cell proliferation of tumor cell lines” were down-regulated (activation z-score, −3.156 to −5.912) (Table 1). These results were consistent with those of the cancer function, proliferation, cell cycle, apoptosis, and migration/invasion assays.

**Table 1:** Gene ontology using IPA software.

Diseases or Functions	*p* value(−log)	Predicted activation	Activation z-score	Number of molecules
Cell movement	16.42	Decreased	−5.912	635
Invasion of cells	15.68	Decreased	−4.633	293
Migration of cells	14.82	Decreased	−5.342	564
Angiogenesis	12.45	Decreased	−3.227	295
Migration of tumor cell lines	11.93	Decreased	−5.019	241
Cell movement of tumor cell lines	11.63	Decreased	−5.602	286
Abnormality in limbs	11.50		−1.888	112
Development of vasculature	11.17	Decreased	−3.227	322
Vasculogenesis	10.64	Decreased	−3.18	243
Benign lesion	10.47		0.922	320
Invasion of tumor cell lines	10.08	Decreased	−4.777	216
Quantity of cells	10.06	Decreased	−5.065	498
Cell proliferation of tumor cell lines	9.95	Decreased	−4.911	452
Abnormal morphology in body cavities	9.82			330
Necrosis of epithelial tissue	9.66		−0.081	189
Apoptosis	9.61		1.486	691
Development of the body trunk	9.51		−1.3	326
Development of epithelial tissue	9.45		−1.765	167
Growth of lesions	9.20	Decreased	−3.186	250
Benign neoplasia	9.20		0.404	286

### Pathway analysis and molecular mechanisms regulated by TRPV2 in ESCC cells

**Figure 3:**
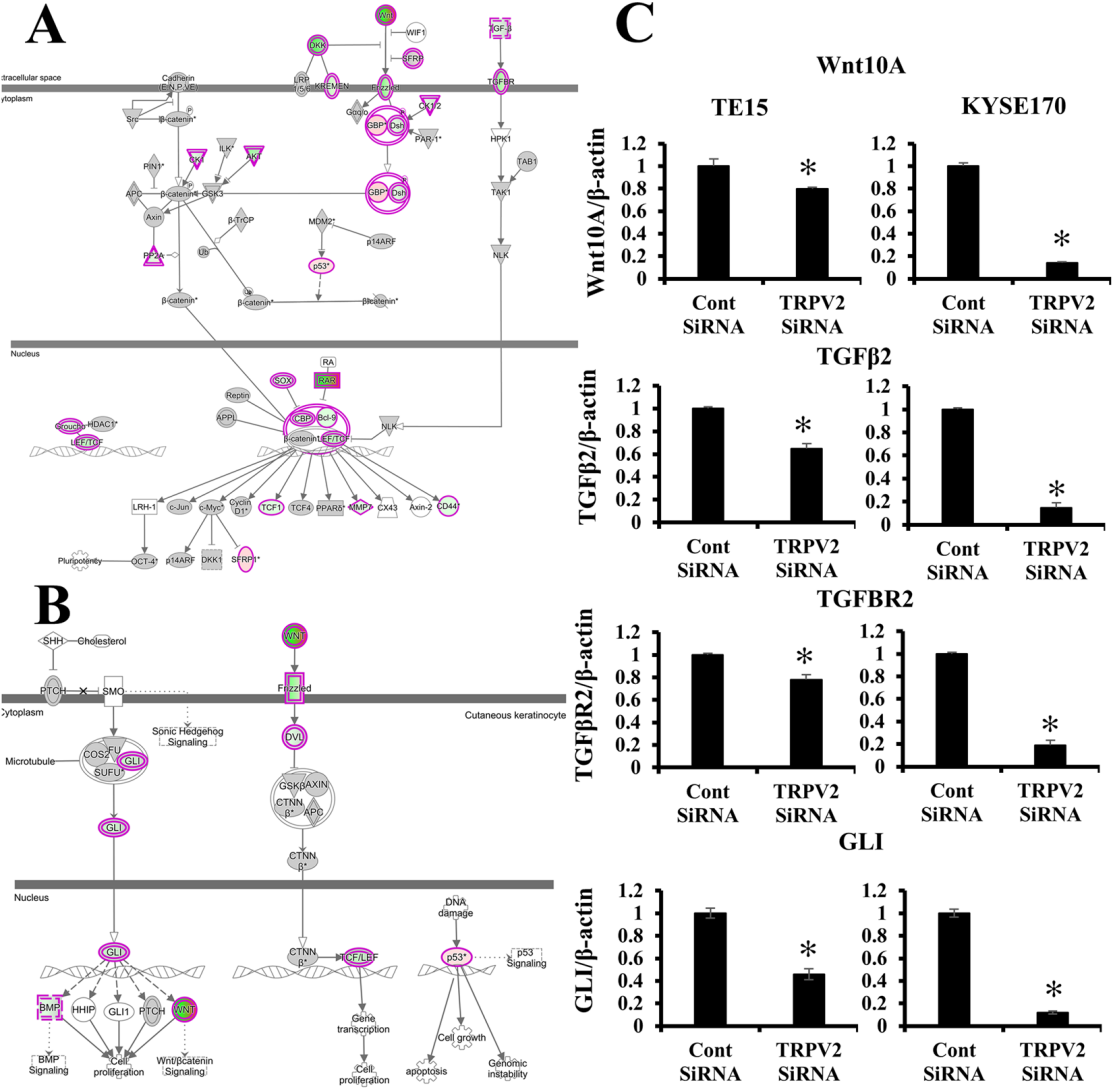
Signal pathways regulated by TRPV2 in ESCC cells. (**A**) (**A**) The signaling map, which was generated using an Ingenuity Pathway Analysis (IPA) software (Ingenuity Systems, Qiagen, Redwood City, CA), of “WNT/β-Catenin”, the top-ranking canonical pathway related to TRPV2 depletion according to an IPA analysis. Red and green indicate genes with expression levels that were higher or lower, respectively, than reference RNA levels. (**B**) The signaling map, which was generated by IPA software, of “Basal cell carcinoma signaling” (**C**) Verification of gene expression by real-time quantitative RT-PCR. The expression levels of four selected “WNT/TGF-βCatenin” and “Basal cell carcinoma signaling”-related genes (WNT10A, TGF-β2, TGF-β2R, and GLI) in TRPV2-depleted TE15 and KYSE170 were compared to those in control siRNA-transfected cells using real-time quantitative RT-PCR. Mean ± SEM. n = 3. *p < 0.05 (significantly different from control siRNA).

We examined the signal transduction networks induced by the TRPV2 knockdown using IPA. The results obtained are shown in Table 2. “WNT/β-Catenin signaling”, “basal cell Carcinoma signaling”, and “regulation of the Epithelial-Mesenchymal Transition Pathway” were the top-ranking canonical pathways related to TRPV2 depletion, while “basal cell carcinoma signaling” and “WNT/β-Catenin signaling” were predicted to be down-regulated pathways (activation z-score: −1.886 and −1.441) (Fig. 3A,B and Supplementary Fig. 1A). Moreover, the pathways associated with stemness, “Human Embryonic Stem Cell Pluripotency”, “Role of NANOG in Mammalian Embryonic Stem Cell Pluripotency”, and “Wnt/β-catenin Signaling”, were the top ranked in the canonical pathway.

**Table 2:** Pathway analysis using IPA software.

Ingenuity Canonical Pathways	*p* value (−log)	Predicted Activation	Activated z-score
Human Embryonic Stem Cell Pluripotency	5.43		
Regulation of the Epithelial-Mesenchymal Transition Pathway	4.29		
Role of Osteoblasts, Osteoclasts and Chondrocytes in Rheumatoid Arthritis	4.26		
Role of Tissue Factor in Cancer	4.03		
Axonal Guidance Signaling	3.96		
Basal Cell Carcinoma Signaling	3.77	Decreased	−1.886
Role of JAK1 and JAK3 in γc Cytokine Signaling	3.77		
UVA-induced MAPK Signaling	3.45	Decreased	−2.065
Role of NANOG in Mammalian Embryonic Stem Cell Pluripotency	3.32	Decreased	−2
Glioblastoma Multiforme Signaling	3.21	Decreased	−2.92
Wnt/β-catenin Signaling	3.04	Decreased	−1.441
Molecular Mechanisms of Cancer	3.01		
Ovarian Cancer Signaling	2.85	Decreased	−2.183
IL-4 Signaling	2.74		
ERK5 Signaling	2.55	Decreased	−2.828
Colorectal Cancer Metastasis Signaling	2.52	Decreased	−3.221
Growth Hormone Signaling	2.46	Decreased	−1.342
Acute Myeloid Leukemia Signaling	2.32	Decreased	−1.706
p53 Signaling	2.32	Increased	0.209
Phospholipases	2.28

In order to confirm the results of the microarray analysis, the expression of several genes included in “WNT/β-Catenin signaling”, “basal cell carcinoma signaling”, “regulation of the Epithelial-Mesenchymal Transition Pathway”, and the relationship between stem cell-related genes and TRPV2 expression were validated in more detail using quantitative RT-PCR. WNT10A, TGFβ2, TGFβR2, GLI, Snai1, Zeb2, CDH2, CD44, and SOX2 mRNA expression levels were lower in TRPV2-depleted TE5 or KYSE170 than in control siRNA-transfected cells (Fig. 3C, Supplementary Figs 1B and 2). These results suggesting that TRPV2 regulated cancer cell function via “WNT/β-Catenin signaling”, “basal cell Carcinoma signaling”, “regulation of the Epithelial-Mesenchymal Transition Pathway” and the expression of stem cell-related genes.

### TRPV2 protein expression in human ESCC samples

**Figure 4:**
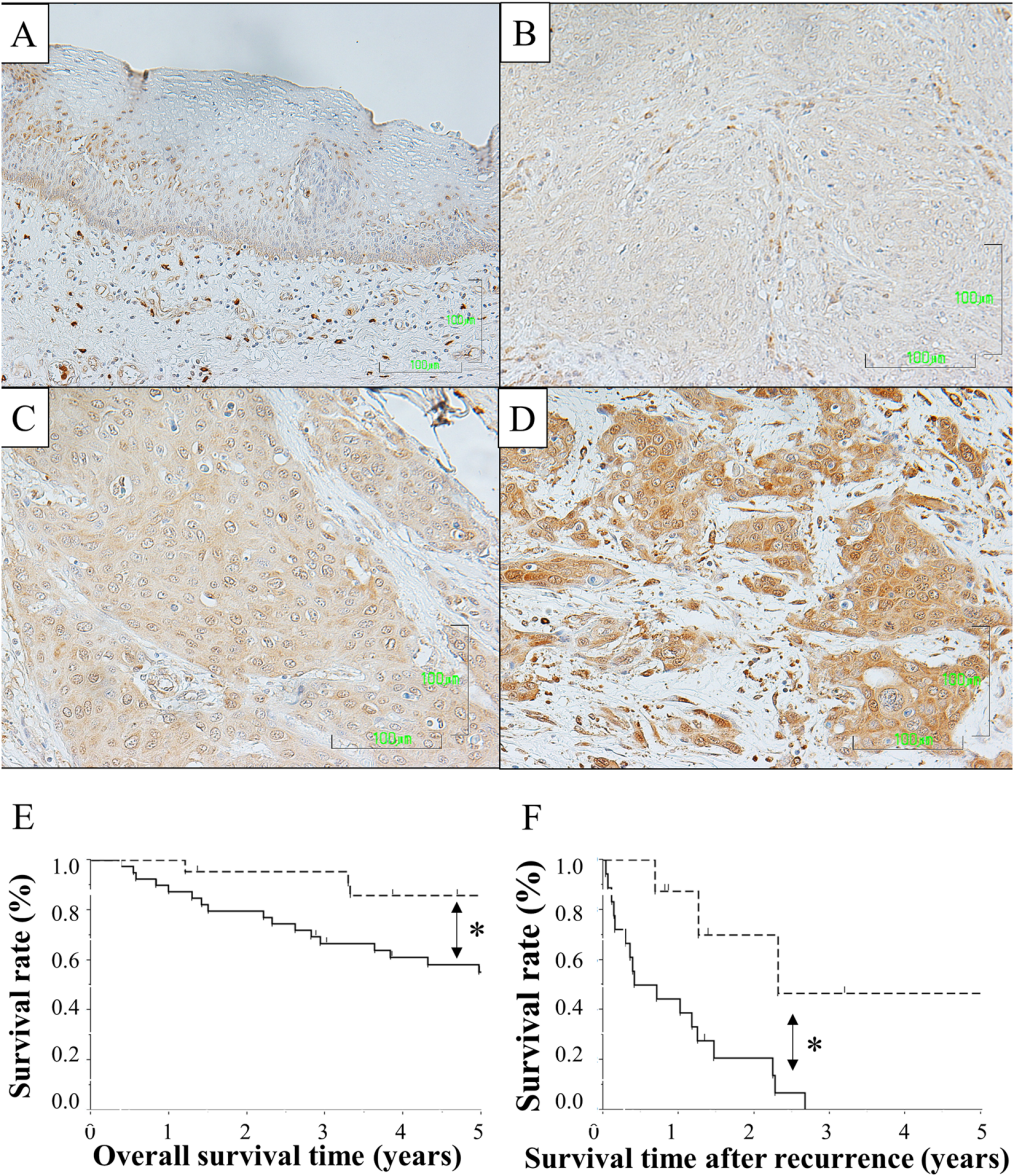
TRPV2 protein expression in human ESCCs. (**A**) Immunohistochemical staining of primary ESCC samples showed that nuclear TRPV2 expression was detected in the middle layer of the non-cancerous stratified squamous epithelium. Magnification: ×400. Bar, 100 μm. (**B**) Immunohistochemical staining of primary human ESCC samples without TRPV2 expression. Magnification: ×400. Bar, 100 μm. (**C**) Immunohistochemical staining of primary human ESCC samples with the weak cytoplasmic expression of TRPV2. Magnification: ×400. Bar, 100 μm. (**D**) Immunohistochemical staining of primary human ESCC samples with the strong cytoplasmic expression of TRPV2. Magnification: ×400. Bar, 100 μm. (**E**) Survival curve of patients after curative resection for ESCC according to the expression of TRPV2. All patients were classified into two groups according to the proportion of strong TRPV2 expression in ESCC tumors: low group: <20%, high group: ≥20%: the low group (n = 22) and high group (n = 40) in the tumor. *p < 0.05: Log-rank test. (**F**) Survival curve of ESCC patients after post-operative recurrence (n = 26) according to the expression of TRPV2. All patients were classified into two groups according to the proportion of strong TRPV2 expression in ESCC tumors: low group: <20%, high group: ≥20%: the low group (n = 8) and high group (n = 18) in the tumor. *p < 0.05: Log-rank test.

In order to evaluate the significance of TRPV2 protein expression in ESCC samples, we performed immunohistochemical staining on 62 tumor samples of Human ESCC using the TRPV2 antibody. Nuclear TRPV2 expression was detected in the middle layer of the non-cancerous stratified squamous epithelium. (Fig. 4A). Although TRPV2 cytoplasmic expression in carcinoma cells varied widely between each ESCC sample, we initially evaluated TRPV2 signal intensities in cancer cells as no, weak, and strong expression (Fig. 4B–D); the proportion of each intensity in tumors was subsequently measured. ESCC samples in which the proportion of strong intensity was 20% or larger were defined as high expression (n = 40), and the others as low expression (n = 22). The relationships between TRPV2 protein expression and patient backgrounds and various clinicopathological parameters were analyzed (Table 3). Tumor sizes were slightly larger in the high group than in the low group. No significant difference was observed in recurrence rate; however, distant recurrence was tended to occur more frequently in TRPV2 high group. Furthermore, the number of cases who underwent radical treatment for recurrence, such as chemoradiotherapy or surgery, was tended to be larger in TRPV2 low group (50.0% vs 38.8%).

**Table 3:** Correlations between clinicopathological features and TRPV2 expression.

		Low group (n = 22)	High group (n = 40)	*p* value
Sex	Male	18	36	0.367
	Female	4	4	
Age	<65	14	21	0.221
	≥65	8	19	
Histology type	Well/Moderate	18	27	0.217
	Poor	4	13	
Location	Ce-Ut	4	5	0.432
	Mt-Lt	18	35	
Tumor size (mm)	<50	17	25	0.082
	≥50	3	14	
Lymphatic invasion	Negative	10	19	0.877
	Positive	12	21	
Venous invasion	Negative	15	20	0.164
	Positive	7	20	
pT	pT1	13	18	0.287
	pT2–4	9	22	
pN	pN0	11	19	0.851
	pN1–3	11	21	
Recurrence	All	8	18	0.509
	Local recurrence	4	6	0.744
	Distant recurrence	4	12	0.150
Initial treatment for recurrence	Radical treatment	4	4	

Ce: cervical esophagus, Ut: upper thoracic esophagus, Mt: middle thoracic esophagus, Lt: lower thoracic esophagus.

pT: pathological tumor invasion depth, pN: pathological lymph node metastasis, Radical treatment: surgery or chemoradiotherapy.

The relationship between the expression of TRPV2 and prognosis of ESCC patients was investigated. The 5-year overall survival rate after surgery was significantly worse in the high group than in the low group (59.5% vs 85.2%, *p* = 0.020) (Fig. 4E). Moreover, the 5-year survival of ESCC patients after post-operative recurrence in the high group was significantly worse than in the low group (46.6% vs 0.0%, *p* = 0.006) (Fig. 4F). A univariate analysis of survival (5-year overall survival) showed that pathological venous invasion, pathological lymph node metastasis, and the strong expression of TRPV2 were significant. A multivariate analysis with these 3 factors (Cox’s proportional hazard model) revealed that the strong expression of TPRV2 was an independent poor prognostic factor (Table 4).

**Table 4:** Prognostic factors of esophageal squamous cell carcinoma according to univariate and multivariate analyses.

		n	Univariable		Multivariable		*p* value
			5-year OS	*p value*	HR	95% CI	
Sex	Male	54	62.9%	0.199			
	Female	8	87.5%				
Age	<65	33	65.9%	0.939			
	≥65	29	66.6%				
Histology type	Well/Moderate	45	71.5%	0.156			
	Poor	17	52.9%				
Lymphatic invasion	Negative	29	70.1%	0.522			
	Positive	33	62.3%				
Venous invasion	Negative	35	78.9%	0.012	2.437	0.983–6.576	0.054
	Positive	27	49.3%				
pT	pT1	31	73.1%	0.165			
	pT2–4	31	59.4%				
pN	pN0	30	79.7%	0.041	2.294	0.915–6.511	0.077
	pN1–3	32	53.6%				
TRPV2 expression	Low group	22	85.2%	0.020	3.153	1.041–13.638	0.041
	High group	40	59.5%				

pT: pathological tumor invasion depth, pN: pathological lymph node metastasis.

## Discussion

A role for TRPV2 in cellular development or morphology was recently reported. Kojima *et al*. showed that TRPV2 was associated with cell cycle progression via the regulation of its translocation induced by Insulin-Like Growth Factor 1^22^. TRPV2 has been shown to play a role in cellular migration through the regulation of intracellular Ca^2+^ concentrations^11^. In the field of oncology, many researchers reported that TRPV2 similarly regulated cell death in cancer cells or cancer migration/invasion^13,15,16,18,23^. They showed that the regulation of Ca^2+^ signaling by TRPV2 may affect these cancer functions. Ca^2+^ is an essential element for the survival and function of cells. Amplifications in the magnitude and duration of intracellular Ca^2^ changes may mean the difference between cellular migration and cell death. In malignant cells, calcium signaling plays important roles in proliferation, apoptosis, tumor stromal interaction, metastasis, and drug resistance^24,25^. In the present study, TRPV2 expression was firstly evaluated, and TRPV2 knockdown experiment was subsequently performed. Although TRPV2 expression in ESCC cell lines was observed, the discrepancy existed between the protein and mRNA expression. Zhang *et al*. described that the intensity of protein expression was not consistent with mRNA expression in over two-third of molecules which expressed in human colorectal cancer specimens^26^. TRPV2 may be one of the molecules with the inconsistency between gene and protein expression. Knockdown experiments demonstrated that TRPV2 depletion suppressed tumor proliferation, cell cycle progression, and migration/invasion, and also induced apoptosis in ESCC cells (Figs 1 and 2). Moreover, the gene ontology analysis revealed that cancer functions, such as cell invasion, angiogenesis, cell migration, cell proliferation, and apoptosis, were down-regulated in TRPV2-depleted ESCC cells (Table 1). These results were consistent with the previously reported antitumor effects induced by the regulation of Ca^2+^ signaling. Therefore, it is plausible that TRPV2 regulates cancer biology via calcium signaling in ESCC.

Furthermore, we performed a pathway analysis to clarify the role of TRPV2 in the cancer signaling of ESCC, and revealed that the depletion of TRPV2 down-regulated “basal cell carcinoma signaling”. “Basal cell carcinoma signaling” is a pathway related to proliferation or apoptosis in basal cell carcinoma, in which cross talk between the hedgehog pathway and Wnt/β-Catenin signaling activates several cancer functions^27,28^. The involvement of the hedgehog pathway in ESCC was previously reported in our laboratory^29^. The present results indicated that TRPV2 regulated malignant potentials via cross talk between the hedgehog pathway and Wnt/β-catenin signaling; furthermore, Ca^2+^ may act as a second messenger between TRPV2 expression and these pathways. Previous studies revealed that intracellular Ca^2+^ plays an important role in the WNT pathway (WNT/calcium pathway)^30,31^. In this pathway, intracellular Ca^2+^ act as a second messenger, resulting in the control of cancer-related gene expression. These results and previous findings suggested that TRPV2 controls WNT/β catenin signaling and basal cell carcinoma signaling (cross talk between the hedgehog and WNT pathways) via the regulation of Ca^2+^ signals, such as WNT/calcium signaling.

TRPV2 depletion also down-regulated “Wnt/β-catenin signaling” in the pathway analysis, which regulated pluripotency via the translocation of β-catenin into the nucleus. The relationship between this pathway and cancer stem cells has already been reported^32,33^. In the microarray data obtained in the present study, TRPV2 depletion down-regulated the expression of the stem cell markers SOX2 and CD44. Furthermore, the top-ranked pathway contained the stemness-related signals “Human Embryonic Stem Cell Pluripotency” and “Role of NANOG in Mammalian Embryonic Stem Cell Pluripotency”. The validation of gene alterations using RT-PCR revealed that CD44 and SOX2 were down-regulated in TRPV2-depleted ESCC cells. We previously reported the overexpression of TRPV2 in cancer stem cells derived from ESCC cell lines, and the present results from the pathway analysis were consistent with these findings. Therefore, TRPV2 may also maintain the stemness of ESCC stem cells via these pathways.

TRPV2 depletion down-regulated the gene expression of TGFβ and TGFβR in “Wnt/β-catenin signaling” in contrast to our exceptions. These results may be explained by the TGFβ paradox. TGFβ plays contrasting roles in tumors, acting as a tumor suppressive gene during the first stages of carcinogenesis and as a tumor promoter during the advanced stages of progression^34,35^. Therefore, the paradoxical results of TGFβ and TGFβR were consistent with our hypothesis.

Moreover, the pathway analysis revealed that the TRPV2 knockdown altered the expression of many genes associated with epithelial-mesenchymal transition (Table 2, rank 2). The validation of gene alterations using RT-PCR revealed that TGFβ, WNT, and SNAIL were down-regulated in TRPV2-depleted ESCC cells. These results suggested that TRPV2 controls the migration/invasion ability of ESCC via epithelial-mesenchymal transition.

Finally, we performed immunohistochemical analysis in order to evaluate clinical significance of TRPV2 expression in ESCC samples. The results revealed that TRPV2 expression was significantly associated with overall survival; however, significant correlations between clinicopathological features and TRPV2 expression were not observed. To clarify the reason of bad prognosis in TRPV2 high group, recurrent patterns and survival after recurrence were analyzed, revealing that distant recurrence were more frequently observed in TRPV2 high group and the prognosis after recurrence in TRPV2 high group was significantly worse than in low group. These findings suggested that TRPV2 affected recurrence pattern or therapeutic effect of therapy for recurrence.

In summary, we herein demonstrated that TRPV2 played a role in the proliferation, cell cycle progression, survival, migration, and invasion of ESCC cells. Microarray results showed that TRPV2 markedly affected the expression of genes related to WNT/β-catenin signaling, basal cell carcinoma signaling (cross talk between the hedgehog and WNT/β-catenin pathways), and regulation of the epithelial-mesenchymal transition pathway. The results of the immunohistochemical analysis of ESCC human samples revealed that the strong expression of TRPV2 was a poor prognostic factor in patients with ESCC. Although further investigations are needed, the present results demonstrate that TRPV2 has potential as a poor prognostic biomarker and novel therapeutic target for ESCC.

## Supplementary information

Supplementary Figure

Supplementary Figure

Supplementary Figure

Supplementary Figure

Supplementary Figure

Supplementary Table 1

Supplementary Table 2

## Data Availability

The data sets generated during and/or analysed during the current study are available from the corresponding author on reasonable request.
