# Supplementary figures and images for: The expression and role of TRPV2 in esophageal squamous cell carcinoma
Source: Sci Rep. 2019 Nov 5;9:16055. doi: 10.1038/s41598-019-52227-0 (PMC6831681; doi:10.1038/s41598-019-52227-0)

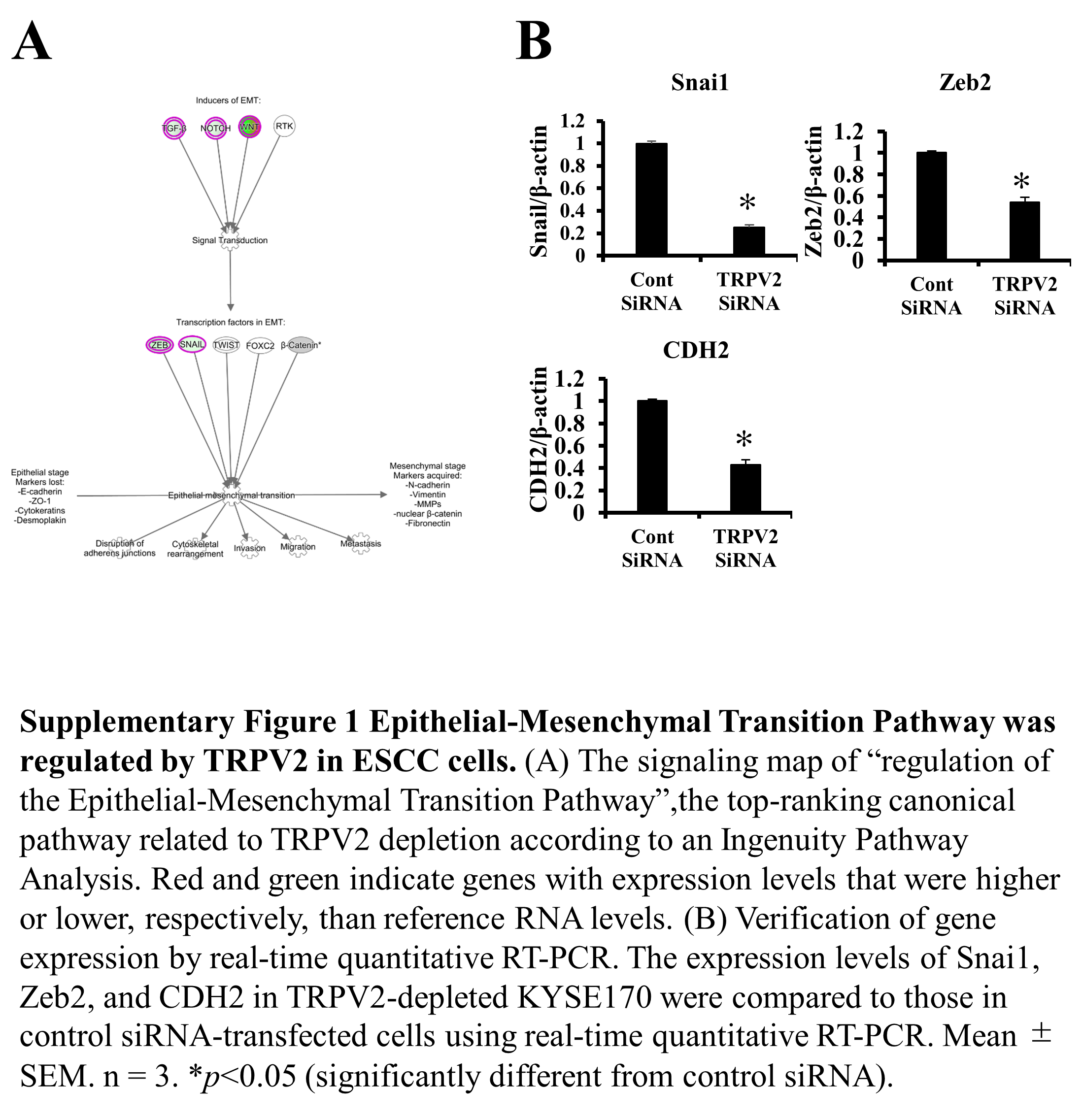

Supplement: Supplementary file 1 — Supplementary Figure [file 41598_2019_52227_MOESM1_ESM.tif]

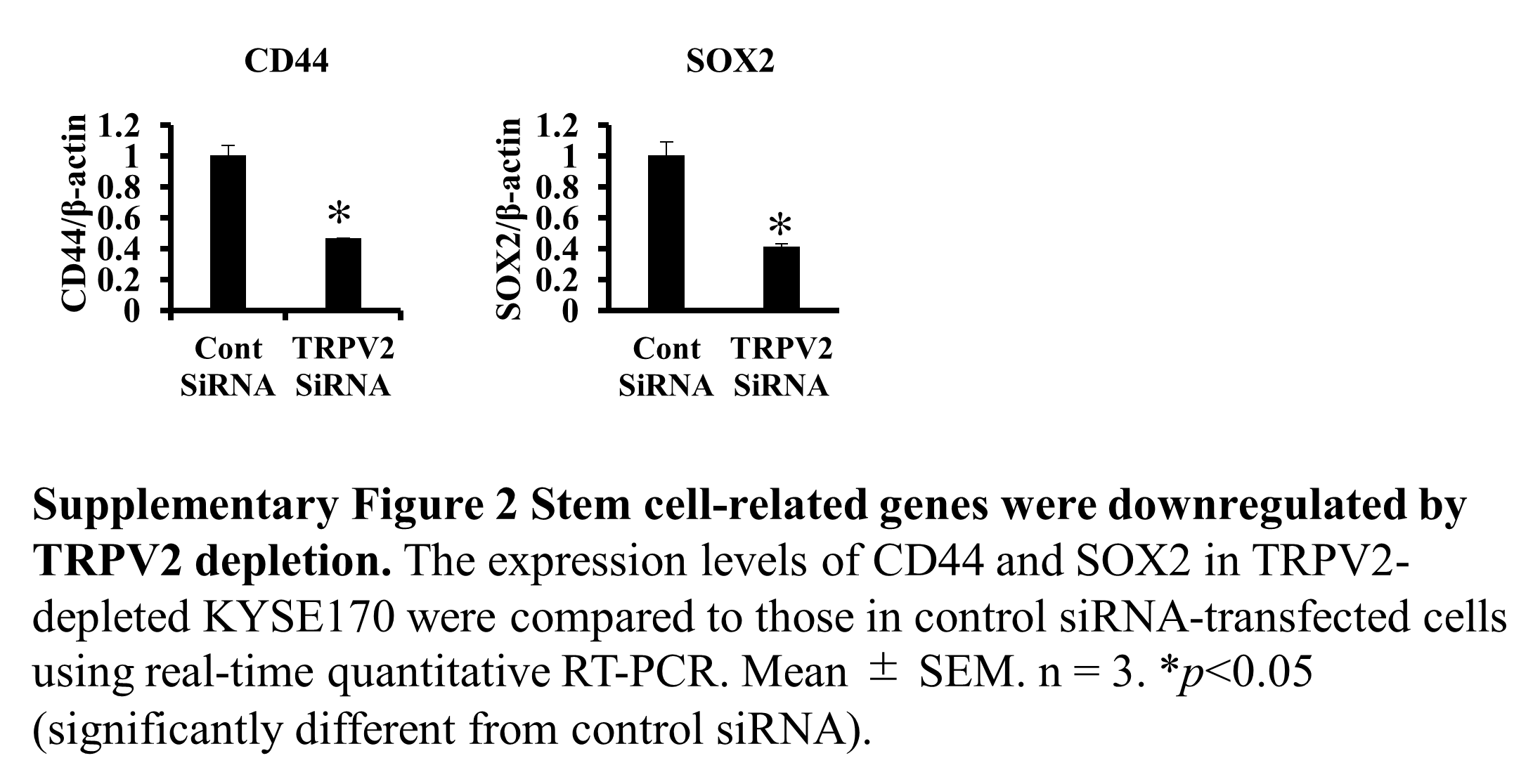

Supplement: Supplementary file 2 — Supplementary Figure [file 41598_2019_52227_MOESM2_ESM.tif]

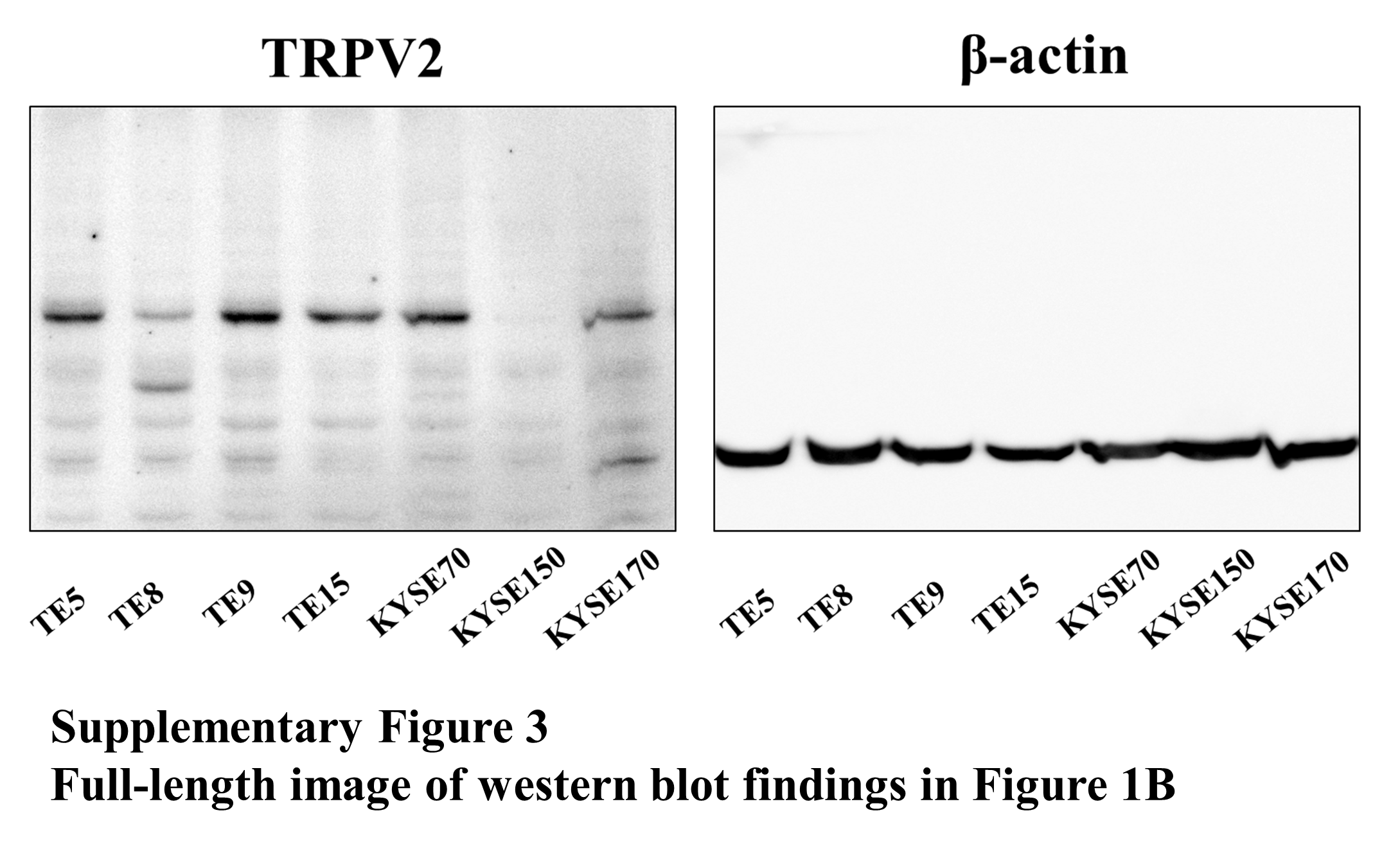

Supplement: Supplementary file 3 — Supplementary Figure [file 41598_2019_52227_MOESM3_ESM.tif]

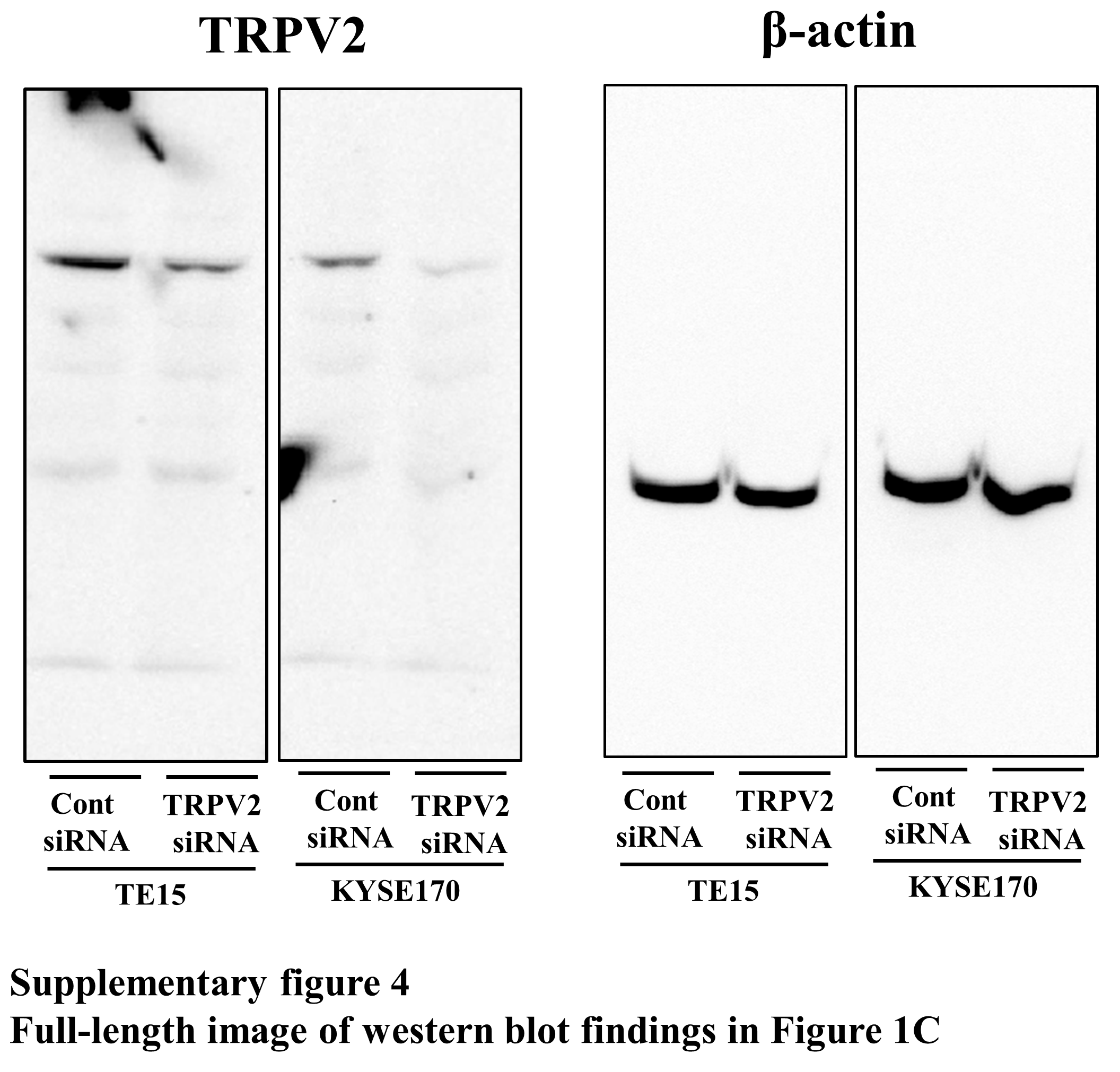

Supplement: Supplementary file 4 — Supplementary Figure [file 41598_2019_52227_MOESM4_ESM.tif]

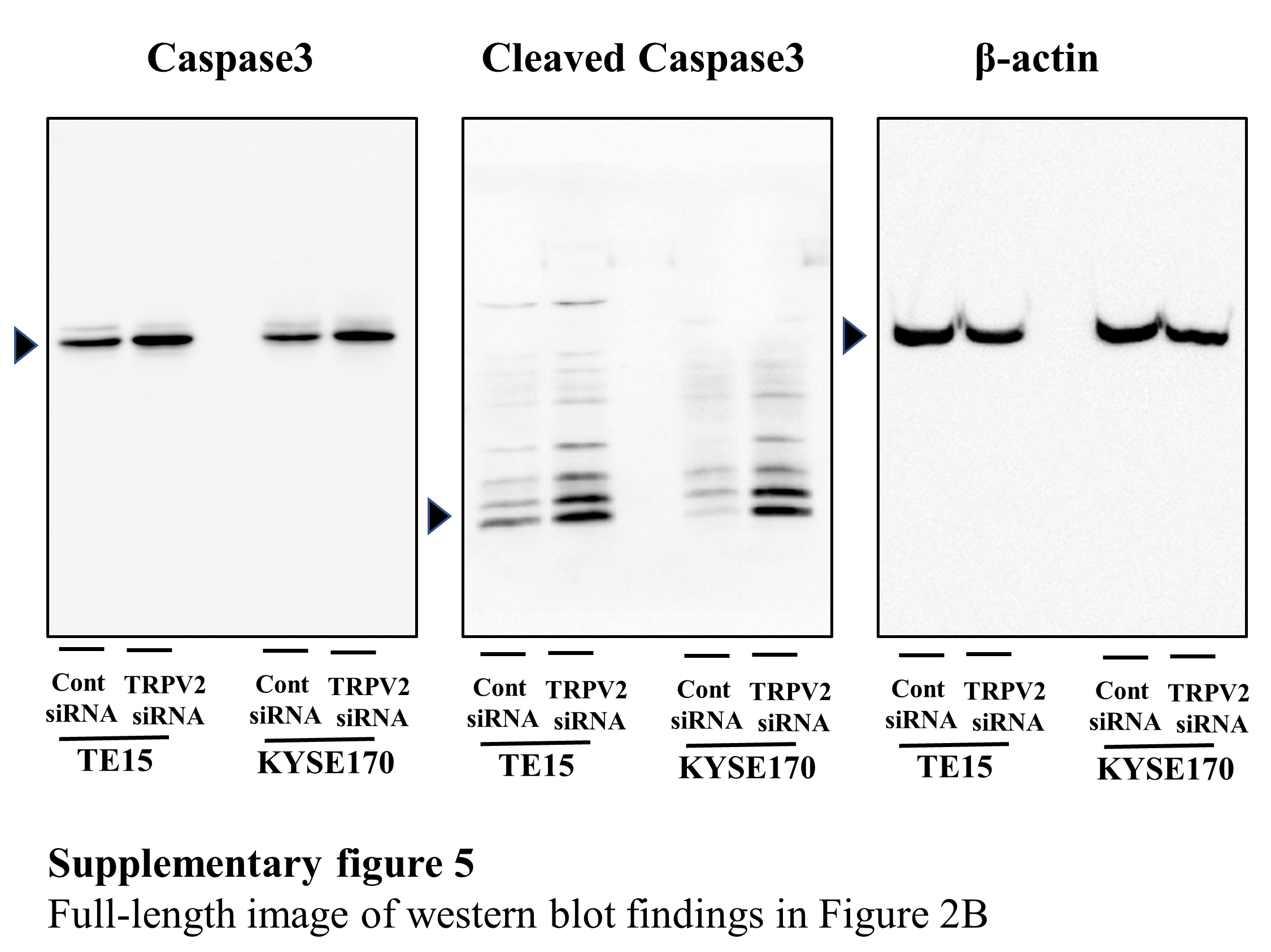

Supplement: Supplementary file 5 — Supplementary Figure [file 41598_2019_52227_MOESM5_ESM.tif]
