# Supplementary material for: The expression and role of TRPV2 in esophageal squamous cell carcinoma
Source: Sci Rep. 2019 Nov 5;9:16055. doi: 10.1038/s41598-019-52227-0 (PMC6831681; doi:10.1038/s41598-019-52227-0)
Supplement: Supplementary file 6 — Supplementary Table 1 [file 41598_2019_52227_MOESM6_ESM.docx]

**Supplementary Table 1 Up-regulated genes in TRPV2-depleted KYSE170 cells**

| Symbol | Gene Name | Exp Fold Change |
| --- | --- | --- |
| MYB | MYB proto-oncogene, transcription factor | 72.128 |
| RNF128 | ring finger protein 128, E3 ubiquitin protein ligase | 70.962 |
| GRM3 | glutamate metabotropic receptor 3 | 70.886 |
| PECAM1 | platelet and endothelial cell adhesion molecule 1 | 63.571 |
| GPC6 | glypican 6 | 60.236 |
| EPPIN | epididymal peptidase inhibitor | 60.041 |
| MSR1 | macrophage scavenger receptor 1 | 59.823 |
| EGOT | eosinophil granule ontogeny transcript (non-protein coding) | 59.045 |
| TAF15 | TATA-box binding protein associated factor 15 | 56.177 |
| ITIH1 | inter-alpha-trypsin inhibitor heavy chain 1 | 50.978 |
| SEZ6L | seizure related 6 homolog like | 50.851 |
| LOXHD1 | lipoxygenase homology domain 1 | 49.746 |
| NTRK1 | neurotrophic receptor tyrosine kinase 1 | 48.346 |
| SMPD5 | sphingomyelin phosphodiesterase 5 | 48.093 |
| OTOG | otogelin | 46.247 |
| TARP | TCR gamma alternate reading frame protein | 45.872 |
| IL17C | interleukin 17C | 41.838 |
| OR8K3 | olfactory receptor family 8 subfamily K member 3 (gene/pseudogene) | 38.363 |
| GMNC | geminin coiled-coil domain containing | 37.221 |
| SLC38A3 | solute carrier family 38 member 3 | 36.641 |
