# Supplementary material for: The expression and role of TRPV2 in esophageal squamous cell carcinoma
Source: Sci Rep. 2019 Nov 5;9:16055. doi: 10.1038/s41598-019-52227-0 (PMC6831681; doi:10.1038/s41598-019-52227-0)
Supplement: Supplementary file 7 — Supplementary Table 2 [file 41598_2019_52227_MOESM7_ESM.docx]

**Supplementary Table 2 Down-regulated genes in TRPV2-depleted KYSE170 cells**

| Symbol | Gene Name | Exp Fold Change |
| --- | --- | --- |
| KRT25 | keratin 25 | -100.542 |
| GLRA4 | glycine receptor alpha 4 | -73.919 |
| MICU1 | mitochondrial calcium uptake 1 | -73.788 |
| OR6C75 | olfactory receptor family 6 subfamily C member 75 | -70.806 |
| AIFM2 | apoptosis inducing factor, mitochondria associated 2 | -70.223 |
| DOCK8 | dedicator of cytokinesis 8 | -61.463 |
| FAM167A | family with sequence similarity 167 member A | -56.625 |
| KLF6 | Kruppel like factor 6 | -53.837 |
| CYP4F22 | cytochrome P450 family 4 subfamily F member 22 | -53.419 |
| ONECUT1 | one cut homeobox 1 | -50.37 |
| KIF26B | kinesin family member 26B | -49.841 |
| OR10G8 | olfactory receptor family 10 subfamily G member 8 | -49.491 |
| ANO5 | anoctamin 5 | -48.903 |
| OR2F1 | olfactory receptor family 2 subfamily F member 1 (gene/pseudogene) | -47.362 |
| CDO1 | cysteine dioxygenase type 1 | -43.046 |
| MAML3 | mastermind like transcriptional coactivator 3 | -41.663 |
| SMPX | small muscle protein, X-linked | -41.215 |
| MLANA | melan-A | -40.094 |
| KRTAP16-1 | keratin associated protein 16-1 | -39.479 |
| CACNB2 | calcium voltage-gated channel auxiliary subunit beta 2 | -37.197 |
